# Study on the Reasonability of Single-Objective Optimization in Miniscrew Design

**DOI:** 10.3390/ma18050973

**Published:** 2025-02-21

**Authors:** Yu-Ching Li, Jiun-Ren Hwang, Chin-Ping Fung

**Affiliations:** 1 Department of Mechanical Engineering, National Central University, Taoyuan 320317, Taiwan; liyuchingbigboss@gmail.com; 2 Department of Mechanical Engineering, Asia Eastern University of Science and Technology, New Taipei City 220303, Taiwan; cpfung@mail.aeust.edu.tw

**Keywords:** miniscrews, optimized design, Taguchi method, stability

## Abstract

Miniscrews are used in orthodontic treatment and can be applied immediately after implantation, making their initial stability crucial. However, clinical reports show that the success rate is not 100%, and many researchers have tried to identify the factors influencing success and optimize designs. A review of the literature reveals that studies on the same geometric parameter of miniscrews using different indicators and different brand samples have led to conflicting results. This study will use consistent miniscrew conditions to verify whether the design differences in the literature are reasonable. This study employs the Taguchi method and ANOVA for optimization analysis. The four control factors comprise thread pitch, thread depth, tip taper angle, and self-tapping notch. Using an L9(3^4^) orthogonal array, the experimental models are reduced to nine. The primary stability indicators for the miniscrew include bending strength, pull-out strength, insertion torque, and self-tapping performance. The results of the single-objective experiments in this study align with the findings from the other literature. However, when analyzed collectively, they do not yield the same optimal solution. Under equal weighting, the combined multi-objective optimal solution is A2B2C1D1. This study exhibits minimal experimental error, ensuring high analytical reliability. The findings confirm that the optimal design does not converge across four single-objective analyses, as different stability indicators yield contradictory trends in design parameters. Given that these four indicators already demonstrate notable discrepancies, the influence of additional stability factors would be even more pronounced. Therefore, a multi-objective optimization approach is essential for the rational design of miniscrews.

## 1. Introduction

Miniscrews have become a mainstream auxiliary device in contemporary orthodontic treatment. These devices can be used immediately after insertion, eliminating the need for a waiting period for osseointegration [1]. By providing either direct or indirect force stability, miniscrews have proven to be effective anchorage solutions within both the mandible and maxillary bone.

Current studies indicate that the miniscrews used in clinical treatments achieve a success rate of approximately 80% [2,3,4,5,6,7]. There are numerous clinical factors that influence the success rate of miniscrew implantation. Human-related factors include bone quality, clinician expertise, smoking habits, oral hygiene, implantation site, and proximity to dental roots. Additionally, there are performance-related factors of miniscrews, such as geometric design, manufacturing process, material selection, and biocompatibility [2,3,4,5,6,7].

In the literature, the human-related factors are typically analyzed using clinical statistical data to refine miniscrew design. In contrast, the performance-related factors, which are directly linked to miniscrew design, are usually investigated through animal experiments, mechanical testing, and simulation analysis. Among these methods, mechanical testing is considered to have the lowest experimental error due to the controlled nature of the testing protocol, miniscrew samples, and artificial bone. Consequently, mechanical testing is the most frequently employed approach in the literature for studying optimal miniscrew design. For the reasons mentioned above, researchers have also explored the mechanical and physical properties of miniscrews in relation to their success rates [8,9]. For instance, Ankit et al. [10] examined the impact of variables such as geometry, materials, dimensions, and loading conditions on miniscrew performance.

Previous studies [11,12] have concluded that miniscrew stability is the key indicator of success. The stability of miniscrews is typically categorized into two phases [13,14]. The first phase is primary stability, which is more related to bone properties and the mechanical performance of miniscrews. This arises primarily from the mechanical engagement between the miniscrew and the cortical bone. This type of stability is established immediately upon implantation. The second phase is secondary stability, which is more related to the biocompatibility of miniscrews. This results from the regeneration and remodeling of the surrounding bone and gingival tissue. As the bone regenerates and gradually adheres to the surface of the miniscrew, a process known as osseointegration occurs. Regardless of these phases, miniscrews serve as effective anchorage devices.

Compared to the secondary phase, primary stability is relatively more critical since most miniscrews are used for durations shorter than the time required for complete osseointegration. As shown in the literature [6], over 64% of the successful cases involved the use of miniscrews for less than three months. Similarly, Kuroda et al. [15] reported that 75% of the cases were in this category when miniscrews were loaded immediately after insertion. Consequently, this study focuses on primary stability during the first phase and does not consider factors such as biocompatibility.

There are numerous factors that influence the primary stability of miniscrews [1], which can be categorized into patient-related factors and miniscrew performance factors. Among these, research on miniscrew performance factors is the most extensive. Researchers [16,17,18,19] have demonstrated a strong correlation between miniscrew performance and geometric design, and have sought to identify the optimal design through testing and simulation analysis. However, the geometric parameters of miniscrews are extensive, including factors such as thread pitch, thread diameter, thread length, thread depth, thread angle, thread tip, thread taper, self-tapping notches, and dual-thread. Despite this complexity, most studies incorporate only a limited subset of these parameters in their optimization research.

On the other hand, the primary stability of miniscrews lacks a standardized indicator. Scribante et al. [20] and Kim et al. [21] proposed that stability can be assessed by the bending strength after implantation, with greater resistance indicating superior primary stability. Excessive lateral bending transforms tensile forces into predominantly axial components, thereby increasing the risk of miniscrew dislodgement. da Cunha et al. [22] evaluated stability using pull-out strength, observing a positive correlation between pull-out strength and primary stability, similar to the relationship observed with lateral force resistance. Kang et al. [23] studied implant stability through insertion torque analysis. They found that unstable manual implantation leads to higher torque, which can cause bone resorption and reduce primary stability. Excessive insertion torque may also result in twisting failure. Barros et al. [24] assessed primary stability based on self-tapping performance, highlighting that inadequate self-tapping performance can cause excessive bone damage, compressing the tapping groove and reducing overall stability.

Based on the existing literature, it can be concluded that the choice of different indicators can lead to significant differences in geometric design outcomes. For instance, when using pull-out strength as an indicator, researchers tend to focus on the design of the tooth angle [25]. Similarly, when using insertion torque as an indicator, researchers focus on the design of the diameter, length, and tip of the miniscrew [26,27].

Another conclusion is that using different indicators for the same geometric parameter may yield contrasting results, making it difficult to determine which is more accurate. For example, in the literature using the thread shape factor (TSF: calculated as the relationship between the mean thread depth and the pitch) as a research indicator, Radwan et al. [28] concluded that a decreased TSF led to increased pull-out forces, while Migliorati et al. [29] reported that a larger TSF provided higher primary stability. This issue, identified from the existing literature, has not been specifically addressed by any researcher.

A further question arises as follows: Is the difficulty in direct comparison due to inconsistent miniscrew conditions across studies, such as varying materials and geometric parameters? To resolve these inconsistencies, this study will standardize the conditions for miniscrews and analyze optimal design outcomes using multiple primary stability indicators. Additionally, it will verify whether the discrepancies in optimal designs reported in the existing literature are reasonable.

## 2. Materials and Methods

The research methodology consists of three parts: The first part involves the preparation of miniscrew specimens, with the number of experiment groups determined based on the selected control factors and the Taguchi method. The second part describes the experimental procedures, which include evaluating the miniscrews according to the ASTM specifications and performing custom-designed experiments. The third part focuses on selecting the quality characteristics for the Taguchi experiment and conducting an analysis of variance (ANOVA).

### 2.1. Specimen Preparation

The literature identifies the four geometric parameters most commonly investigated for the impact on the primary stability of miniscrews which are thread pitch, thread depth, thread tip, and self-tapping notch [1,18,22,26,30,31]. Increasing the thread pitch generally aims to facilitate faster insertion of the miniscrew into the bone; however, the literature indicates that a larger thread pitch also increases insertion torque, which may affect the quality of fixation [16]. Increasing the thread depth is intended to enhance bone engagement and improve stability, but it significantly reduces the twist strength of the miniscrew [31]. Sharper thread tip designs enhance initial positioning and penetration but also increase the risk of tip breakage [32,33]. The self-tapping notch is designed to enable direct insertion without pre-drilling, thereby accelerating the procedure [26]; however, it also raises the risk of screw fracture during removal [32]. Given the aforementioned findings, conclusions drawn from studies focusing on individual design parameters are inadequate to elucidate their interactive effects. Therefore, a comprehensive analysis that integrates these four design parameters is essential.

Assuming each of the four parameters has three levels, a total of 81 experimental combinations would be required using the one-factor-at-a-time (OFAT) method. To reduce the number of experiments and obtain robust results, this study employed the traditional Taguchi method with an orthogonal array L9(3^4^), narrowing the experiments to 9 trials, as shown in Table 1. To further define the levels of each parameter, the thread pitch and thread depth were set to the most commonly used values as critical levels. The tip taper angle and self-tapping notch were defined by selecting the maximum and minimum values reported in the literature. As shown in Table 2, the range of the levels was also obtained based on a literature review [1,18,22,26,30,31].

**Figure 1:**
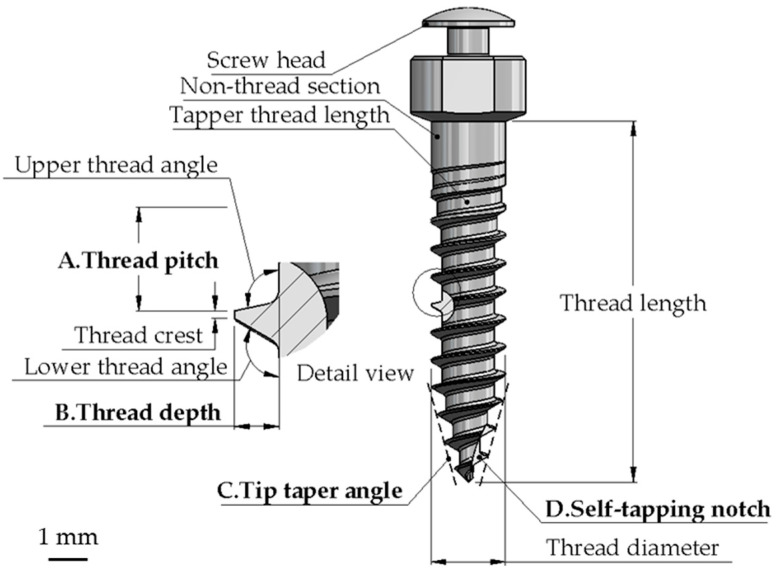
Schematic diagrams of the miniscrews.

In addition to the four control factors discussed above, the other design parameters adhered to the most commonly used specifications or dimensions. These included a thread diameter of 2 mm, a thread length of 10 mm, a non-threaded section of 1 mm, a tapered thread length of 2 mm, an upper thread angle of 102°, a lower thread angle of 120°, a thread crest of 0.05 mm, and a mushroom-shaped screw head. The corresponding locations of these parameters are illustrated in Figure 1.

The miniscrews were fabricated from SUS316LVM (UNS S31673), a medical-grade stainless steel (Zapp AG, Nordrhein-Westfalen, Germany). This material conforms to ASTM F138-19 [34] and ISO 5832-1:2016 [35] standards and exhibits a tensile strength of 1400 MPa. The artificial bone used for experimenting was polyurethane foam (Sawbone, Division of Pacific Research Laboratories, Inc., Vashon, WA, USA), which complies with the ASTM F1839-08 [36] standard and is specifically designed for biomechanical experiments. For this study, the hardest grade, PCF 50, with a density of 0.8 g/cm^3^, was selected to simulate human bone for the relevant experiments.

The production method for the miniscrews involved CNC automatic lathe machining, with the threading section processed using a customized forming tool. All the specimens and testing fixtures were manufactured by a professional company (Bomei Co., Ltd., Taoyuan, Taiwan), which specializes in the production of miniscrew products. All the tolerances and machining conditions were strictly controlled according to the standard operating procedures to minimize sample variability and ensure reproducibility.

### 2.2. Testing Methods and Equipment

The primary stability of miniscrews was evaluated using indicators such as insertion torque, pull-out strength, bending strength, and self-tapping performance. The experiments for insertion torque and pull-out strength were conducted in accordance with ASTM F543-17, specifically sections A2 and A3 [37]. The experiments for bending strength and self-tapping performance were custom-designed.

For measuring insertion torque, a torque testing machine (MODEL 2205S, SE Test-systems Co., Ltd., New Taipei City, Taiwan) equipped with a torque load cell (NTS Technology Co., Ltd., Tokyo, Japan) was adopted. The test block dimensions were 20 mm × 20 mm × 40 mm, and an 11.2 N axial load was maintained on the torque testing machine to ensure the proper engagement of the miniscrew with the test block during the insertion test. The insertion speed was set to 3 rpm in accordance with ASTM F543-17, section A2. The insertion and removal torque values were recorded over the first four rotations after the torque began to register. Additionally, the maximum insertion torque (MIT) and maximum removal torque (MRT) values were also recorded.

The self-tapping performance test employed the same setup as the insertion torque measurement. During the insertion torque test, the initial insertion angle (IIA) required for the torque value to transition from zero to nonzero was simultaneously recorded. A nonzero torque indicated that the tip of the miniscrew had penetrated the surface of the test block. This initial insertion angle served as an indicator of self-tapping performance and was inversely proportional to the self-tapping performance.

The equipment used to measure the pull-out strength and bending strength was an automatic servo vertical testing machine (JSV-H1000, Japan Instrumentation System Co., Ltd., Sakurai-Shi, Japan). According to the ASTM F543-17, section A3 standards, the same test block used for insertion torque measurements was selected. The miniscrews were vertically inserted into the test block at a speed of 3 rpm until 60% of the thread length was inserted. The setup was then mounted on the testing machine using fixtures, and a tensile load was applied at a speed of 5 mm/min until the miniscrew detached from the test block or the pull-out load reached zero. The displacement and pull-out loads were recorded, and the maximum pull-out load was also recorded.

**Figure 2:**
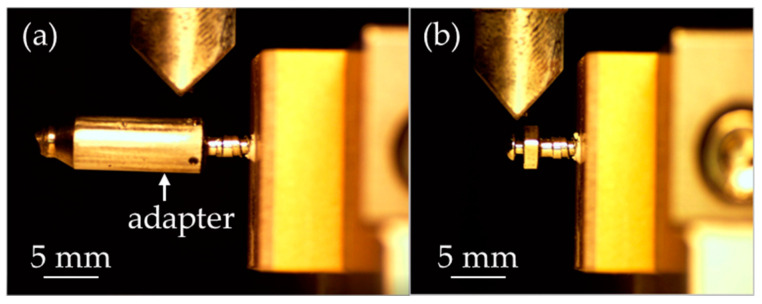
The bending test setup (**a**) with and (**b**) without a cylindrical adapter.

For the bending strength test, the same specimen setup as the pull-out strength test was used, but the sample was positioned horizontally on the testing machine. The testing machine’s press head was fitted with a pointed tip to ensure point contact during the bending test, conducted at a speed of 1 mm/min [21]. A cylindrical adapter was covered over the head of the miniscrew to prevent the irregular shape of the screw head from influencing the results, as shown in Figure 2. The maximum load was recorded during the test.

### 2.3. Taguchi Method’s Quality Characteristics and ANOVA

The testing results were analyzed using the Taguchi method, which calculates the signal–noise ratio (S/N ratio) for various quality characteristics. Analysis of variance (ANOVA) was employed to evaluate the S/N ratios, identify significant control factors, and quantify each factor’s contribution to the objectives. Finally, the main effects plot of the S/N ratios was used to determine the optimal levels of the control factors.

The insertion torque and self-tapping performance were assessed using the “Smaller-the-Better” quality characteristic. Excessive insertion torque may lead to miniscrew fracture or cause instability during manual operation due to lateral wobbling, thereby compromising primary stability. A smaller initial insertion angle during bone insertion indicates superior tapping ability. Conversely, pull-out strength and bending strength were evaluated using the “Larger-the-Better” quality characteristic, as higher experimental values indicate greater primary stability of the miniscrew. Each quality characteristic was tested five times (*n* = 5), and the results were included in the analysis.

## 3. Results

**Figure 3:**
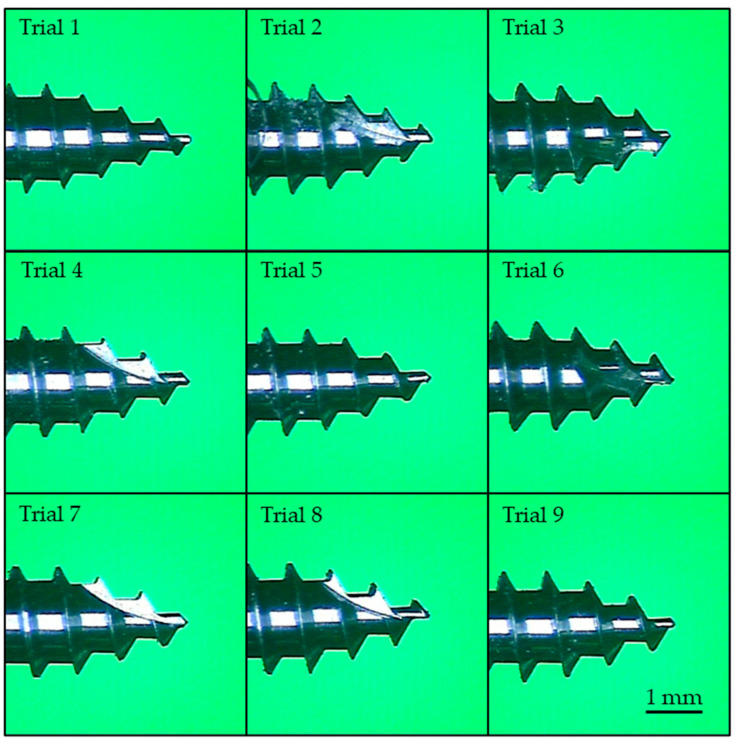
Thread profiles of trials 1 to 9.

Figure 3 illustrates trials 3, 4, and 8, each appearing to have only one self-tapping notch. However, there are actually two self-tapping notches, with the second located on the opposite side of the miniscrew, which is not visible in the photograph. Trials 1, 4, and 7 have the shallowest thread depth of 0.25 mm, while trials 3, 6, and 9 have the deepest thread depth of 0.35 mm. These differences are clearly distinguishable in the images.

**Figure 4:**
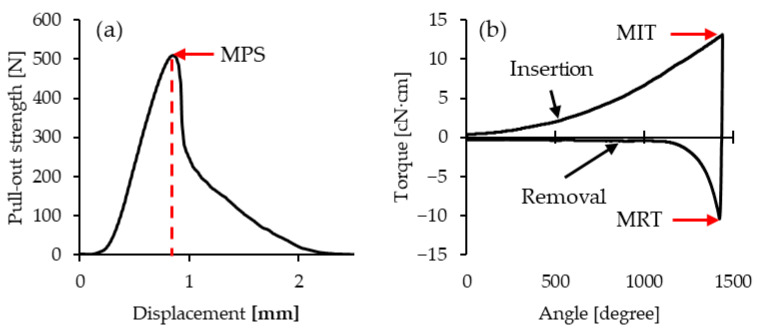
Test results of trial 1 specimen #1. (**a**) Pull-out strength; (**b**) insertion and removal torque.

Trials 3, 5, and 7 exhibit a larger (blunter) taper at the screw tip, though this difference is difficult to discern in the photographs due to the viewing angle and the presence of self-tapping notches. The variations in thread pitch are minimal and cannot be visually identified; measurement is required to quantify these differences. Figure 4 shows the results of the pull-out strength and insertion torque tests for trial 1, specimen #1. Similar trends were observed for the other specimens, with no significant deviations noted.

During the insertion and removal tests, the maximum insertion torque (MIT) for each trial was extracted, and the mean, standard deviation, and S/N ratio were calculated. The results are summarized in Table 3, which also includes the maximum initial insertion angle (MIIA), maximum pull-out strength (MPS), and maximum bending strength (MBS) for each trial.

The standard deviations (SD) for all the trials were less than 5% of the mean values, indicating the high consistency and reproducibility of the experimental results. For MIT, the minimum value was 9.71 cN·cm (trial 3), while the maximum was 15.34 cN·cm (trial 9), representing a 37% difference. The MIIA ranged from 259.54° (trial 8) to 369.04° (trial 7), with a 30% difference. For MPS, the highest value was 560.35 N (trial 5), and the lowest was 435.91 N (trial 3), showing a 22% difference. Regarding MBS, the maximum value was 124.29 N (trial 4), while the minimum was 79.53 N (trial 9), reflecting a 36% difference.

The contributions of control factors were analyzed using ANOVA, as shown in Table 4. The most significant control factor affecting the MIT was thread pitch (44.9%), followed by the self-tapping notch (42.5%). For the MIIA, the tip taper angle was the most influential factor (35.6%), with the self-tapping notch as the second most significant factor (30.3%). The self-tapping notch had the largest contribution to MPS at 66.6%, followed by thread pitch with 19.7%. For the MBS, thread depth was the dominant factor, contributing 91.9%, while the contributions of all the other factors were below 5% and were, therefore, not considered.

**Figure 5:**
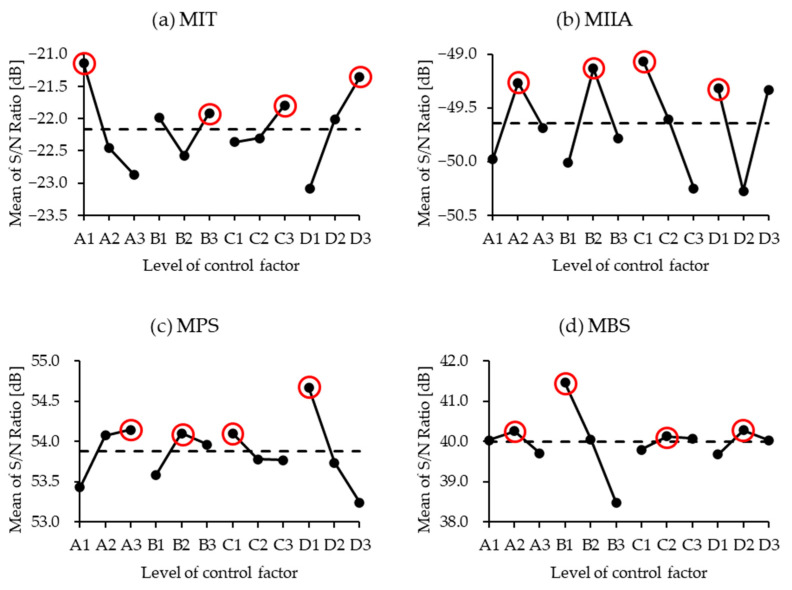
The main effects plot for each control factor. (**a**) MIT: maximum insertion torque; (**b**) MIIA: maximum initial insertion angle; (**c**) MPS: maximum pull-out strength; (**d**) MBS: maximum bending strength.

The main effects plot for different levels of each control factor is presented in Figure 5. The magnitude of the differences among the levels of each control factor in the main effects plot reflects their influence on the results, which is consistent with the findings from the ANOVA analysis. Furthermore, the trends observed in the main effects plot enable the determination of the optimal levels for each control factor.

For single-objective optimization, as indicated by the red circle in Figure 5, achieving a lower MIT corresponds to the optimal combination of the control factors A1B3C3D3 while minimizing the MIIA results in the optimal combination A2B2C1D1. To maximize MPS and MBS, the optimal combinations are A3B2C1D1 and A2B1C2D2, respectively.

## 4. Discussion

The clinical application of miniscrews is associated with a failure rate of approximately 20% [2,3,4,5,6,7], with failure rates reaching up to 40% in certain anatomical locations [38,39]. Despite decades of advancements, the failure rate of miniscrews has not substantially decreased, highlighting the need for continued research and optimization in their design.

In the Taguchi experiment, maintaining the dimensional precision of miniscrews within a trial is critical. In this study, the difference in pull-out strength across the nine trials was 22%. Specifically, when the results were ranked by value, the difference between any two adjacent trials could be as low as 2.75%. Consequently, if the geometric dimensions of miniscrews within a trial vary excessively, the resulting data dispersion may obscure the contributions and levels of control factors, thereby compromising the scientific validity of the experimental results. The low standard deviations observed in this study indicate high reproducibility and confirm that the dimensional precision of the test samples was well controlled with minimal individual variability.

As shown in Figure 4b, the insertion torque curve demonstrates a gradual increase during the initial 1000° of insertion, followed by a more linear trend. This behavior is attributed to the tapered design of the first 3–4 threads of the miniscrew, which results in a curved growth in frictional resistance as they engage with the bone during the initial insertion. Beyond 1000°, the threads become straight, leading to a proportional relationship between torque and insertion depth.

In contrast, the removal torque curve exhibits a sharp drop in torque from 1440° to 1140°, after which resistance becomes nearly negligible. This observation aligns with clinical experience, where loosening the miniscrew significantly reduces the effort required for its removal. The characteristics of both curves are consistent with trends reported in the literature [23,27,40].

The experimental results are organized into Table 4 based on single-objective optimization. For MIT, the thread pitch was found to be the most significant contributing factor. This is because the study used a fixed number of rotations in the experiments; consequently, a smaller thread pitch resulted in a shallower actual insertion depth and a reduced contact area with the artificial bone. The main effects plot revealed that MIT is directly proportional to thread pitch. The optimal condition for achieving a lower MIT was determined to be the smallest thread pitch of 0.75 mm, consistent with the findings reported in the literature [41].

The second most significant factor influencing MIT was the self-tapping notch. A greater number of self-tapping notches creates cutting-edge structures on the threads, making the insertion process more akin to bone scraping with a tool, similar to the concept of pre-drilling [42]. However, an excessive number of self-tapping notches is not always beneficial. As noted in the literature [32], too many notches may lead to increased removal torque after osseointegration due to excessive bone overgrowth around the threads, potentially resulting in miniscrew fracture during removal.

In terms of self-tapping performance, tip tapper angle and self-tapping notch were found to have the greatest influence. It is expected that the sharper tip design will have better self-tapping performance. From the perspective of insertion torque, a higher number of self-tapping notches would theoretically facilitate the formation of pre-drilling holes to guide thread engagement. However, the results from the MIIA experiments did not align with this expectation. Instead, the miniscrews without self-tapping notches demonstrated superior bone drilling performance. Further investigation revealed that an increased number of self-tapping notches reduced the sharpness of the thread features at the tip by approximately half, as shown in Figure 3, subfigure trial 4. This reduction prevented the threads from effectively engaging with the artificial bone, causing the miniscrew to idle within the pre-drilling hole on the surface. These findings provide a novel perspective not previously discussed in related studies [26,42,43,44].

Discussing pull-out strength, the relationship between force and displacement is illustrated in Figure 4a. The MPS is defined as the peak value on the curve, closely resembling the results from experiments using porcine bone [45]. This similarity indicates that the use of artificial bone in the experiments provides a reasonably accurate approximation. The pull-out strength testing was conducted at a consistent insertion depth in this study. Therefore, a higher number of self-tapping notches, similar to the findings on self-tapping performance, leads to incomplete thread engagement, reducing the surface area where the threads can grip the artificial bone during the pull-out test, thereby significantly decreasing the pull-out strength. This observation is further supported by the main effects plot, which confirms that miniscrews without self-tapping notches (D1) provide the best performance. The secondary contributing factor to pull-out strength is thread pitch. Under the same insertion depth, a smaller pitch results in more thread turns, increasing thread engagement with the bone and thereby improving pull-out strength. These findings are consistent with the literature [1,27,41].

Regarding bending strength, since the major diameter is fixed, a greater thread depth results in a smaller minor diameter, reducing the solid core area of the miniscrew and consequently leading to poorer bending strength [20,46,47]. Notably, some studies used miniscrews from different brands. To account for design differences between brands, some studies introduced an additional factor: the ratio of thread depth to maximum major diameter. The impact of the other control factors was minimal; therefore, when discussing single-objective bending strength, it can be neglected.

The contribution of the tip taper angle to the three indicators (MIT, MPS, and MBS) is minimal; therefore, this parameter can be neglected when optimizing the design of the miniscrews. The other three parameters each contribute more than 50% to specific indicators, indicating that single-objective studies in the literature are not necessarily incorrect. They may lack comprehensiveness and objectivity.

From the perspective of single-objective test results, this study’s findings align closely with those of other studies. However, when comparing them collectively, it is not possible to achieve the same optimal solution. For example, the role of the self-tapping notch differs significantly across various objectives. This is similar to the findings of the studies on TSF in the literature. Thus, designing and evaluating miniscrews based on a single objective might result in trade-offs, where some factors are prioritized over others. The results of this study, based on consistent miniscrew conditions, provide a more scientifically grounded explanation for why different studies, despite using the same geometric parameters, present varying conclusions. The discrepancies arise from the selection of different evaluation indicators.

This study validates that the single-objective approach has limitations in optimizing miniscrew design. Even within this study, the four selected indicators yielded differing results. Moreover, the factors influencing miniscrew stability extended far beyond the indicators considered in this research. This highlights the necessity of a multi-objective approach to comprehensively account for the various conditions of miniscrew usage. Currently, there is no reference in the literature regarding the actual weight of the four indicators affecting implantation success rate. Therefore, this study assumes equal weight for all indicators. The factor levels that appear most frequently in the statistical analysis are considered the optimal solution, resulting in A2B2C1D1. The corresponding optimal levels are a thread pitch of 0.8 mm, thread depth of 0.3 mm, tip taper angle of 20.4°, and no self-tapping notches. Since this optimal solution is different from the trial of the experimental design, further prototype testing is required to verify whether it meets 95% confidence intervals.

In addition to the subjective method of this study, several multi-objective statistical methods can be applied to the design of miniscrews that need to satisfy multiple functional requirements. These methods include gray relational analysis [48], principal component analysis [49], linear regression [50], and utility theory [30]. Such approaches provide a more objective framework for optimization design and could serve as the basis for further in-depth research.

## 5. Conclusions

In this study, it is assumed that the miniscrew has not yet fully osseointegrated with the bone. A single-objective optimization study was conducted using mechanical tests reported in the literature to evaluate primary stability. By maintaining consistent conditions for all the parameters except the controlled factors, experimental error was minimized, thereby providing more objective research results. Based on these experiments and subsequent analysis, the following conclusions were drawn:

- The Taguchi method effectively reduces the number of experiments.
- This study confirmed that the same control factor can have opposite effects on different evaluation indicators.
- When researching the optimization design of miniscrews, focusing solely on a single indicator would be unreasonable.
- Multi-objective optimization should be considered to determine the optimal parameters.
- This study provides an optimization result based on equal weighting, which can serve as a valuable reference for manufacturers in designing miniscrews.

## 6. Limitations

When examining conflicting arguments in the literature, this study aims to minimize experimental errors and validate the feasibility of the research methodology. To achieve this, it considers only the four most commonly used mechanical tests as indicators for evaluating miniscrew stability and identifies the optimal results for four key geometric parameters. However, numerous factors influence the stability of miniscrews implanted in bone, as discussed in the introduction. Therefore, the multi-objective results of this study are valid only under the specific conditions of this research. If additional indicators can be quantified and more advanced multi-objective analysis methods validated, they could be incorporated into future studies, thereby enhancing the comprehensiveness and robustness of the results.

## Figures and Tables

**Table 1:** The L9(3^4^) orthogonal array and control factor levels.

Trial	Control Factor Level			
	Thread Pitch [mm]	Thread Depth [mm]	Tip Taper Angle [Degree]	Self-Tapping Notch [Amount]
1	1	1	1	1
2	1	2	2	2
3	1	3	3	3
4	2	1	2	3
5	2	2	3	1
6	2	3	1	2
7	3	1	3	2
8	3	2	1	3
9	3	3	2	1

**Table 2:** Control factors and levels.

Control Factor	Level		
	1	2	3
A. Thread pitch [mm]	0.75	0.8	0.85
B. Thread depth [mm]	0.25	0.3	0.35
C. Tip taper angle [degree]	20.4	24.8	29.1
D. Self-tapping notch [amount]	0	1	2

**Table 3:** The mean, standard deviation, and S/N ratio of the four tests.

Trial	MIT		MIIA		MPS		MBS	
	Mean ± SD	S/N	Mean ± SD	S/N	Mean ± SD	S/N	Mean ± SD	S/N
	[cN·cm]	[dB]	[degree]	[dB]	[N]	[dB]	[N]	[dB]
1	12.75 ± 0.51	−22.11	296.78 ± 08.60	−49.45	509.02 ± 04.92	54.13	112.50 ± 8.23	40.97
2	11.98 ± 0.20	−21.57	318.46 ± 09.01	−50.06	467.84 ± 10.92	53.40	106.34 ± 7.04	40.49
3	09.71 ± 0.16	−19.74	331.40 ± 16.13	−50.42	435.91 ± 24.49	52.75	085.40 ± 2.75	38.62
4	12.06 ± 0.39	−21.63	291.26 ± 12.76	−49.29	448.89 ± 16.31	53.03	124.29 ± 4.56	41.87
5	14.87 ± 0.33	−23.45	282.98 ± 12.02	−49.04	560.35 ± 14.47	54.96	100.73 ± 3.18	40.05
6	13.00 ± 0.15	−22.28	297.12 ± 07.79	−49.46	514.32 ± 06.11	54.22	087.51 ± 4.09	38.82
7	12.87 ± 0.63	−22.20	367.04 ± 10.92	−51.30	477.78 ± 05.40	53.58	119.29 ± 3.05	41.53
8	13.63 ± 0.28	−22.69	259.54 ± 12.03	−48.29	498.02 ± 09.39	53.94	095.54 ± 2.10	39.60
9	15.34 ± 0.28	−23.72	297.34 ± 10.13	−49.47	557.00 ± 19.18	54.90	079.53 ± 1.61	38.01

**Table 4:** The contributions of the control factors in the four tests.

Control Factor	Contribution [%]			
	MIT	MIIA	MPS	MBS
A. Thread pitch	44.9	13.0	19.7	3.0
B. Thread depth	7.2	21.2	9.1	91.9
C. Tip taper angle	5.4	35.6	4.6	1.3
D. Self-tapping notch	42.5	30.3	66.6	3.8

## Data Availability

The original contributions presented in this study are included in the article. Further inquiries can be directed to the corresponding author.

## References

[1] Migliorati M., Benedicenti S., Signori A., Drago S., Barberis F., Tournier H., Silvestrini-Biavati A. (2012). Miniscrew design and bone characteristics: An experimental study of primary stability. Am. J. Orthod. Dentofac. Orthop..

[2] Chang C.H., Lin J.S., Roberts W.E. (2018). Failure rates for stainless steel versus titanium alloy infrazygomatic crest bone screws: A single-center, randomized double-blind clinical trial. Angle Orthod..

[3] Giuliano Maino B., Pagin P., Di Blasio A. (2012). Success of miniscrews used as anchorage for orthodontic treatment: Analysis of different factors. Prog. Orthod..

[4] Lee D., Park J.H., Bay R.C., Choi S., Chae J. (2020). Cortical bone thickness and bone density effects on miniscrew success rates: A systematic review and meta-analysis. Orthod. Craniofacial Res..

[5] Baumgaertel S., Palomo J.M., Zaverdinos M., Elshebiny T. (2020). Ten years of miniscrew use in a U.S. orthodontic residency program. Am. J. Orthod. Dentofac. Orthop..

[6] Song Y.S., Yow M., Chew M.T., Foong W.C., Womg H.C. (2015). A study of success rate of miniscrew implants as temporary anchorage devices in Singapore. Int. J. Dent..

[7] Lam R., Goonewardene M.S., Allan B.P., Sugawara J. (2018). Success rates of a skeletal anchorage system in orthodontics: A retro-spective analysis. Angle Orthod..

[8] Shah A.H., Behrents R.G., Kim K.B., Kyung H.-M., Buschang P.H. (2012). Effects of screw and host factors on insertion torque and pullout strength. Angle Orthod..

[9] Reynders R.A.M., Ronchi L., Ladu L., van Etten-Jamaludin F., Bipat S. (2012). Insertion torque and success of orthodontic mini-implants: A systematic review. Am. J. Orthod. Dentofac. Orthop..

[10] Jedliński M., Janiszewska-Olszowska J., Mazur M., Grocholewicz K., Suárez Suquía P., Suárez Quintanilla D. (2022). How does orthodontic mini-implant thread minidesign influence the stability?—Systematic review with meta-analysis. J. Clin. Med..

[11] Katyal S., Bhatia N.K., Sardana R., Singh S., Chugh A., Shamim M.A., Anil A., Negi A., Chugh V.K. (2024). Success rate and factors affecting stability of infrazygomatic miniscrew implants: A systematic review and meta-analysis. Eur. J. Orthod..

[12] Lee Y., Choi S.-H., Yu H.-S., Erenebat T., Liu J., Cha J.-Y. (2021). Stability and success rate of dual-thread miniscrews. Angle Orthod..

[13] Çaglar S., Özlem A. (2013). Techniques to measure miniscrew implant stability. J. Orthod. Res..

[14] Mutheus M.P., Matilde G.N., Lincolin I.N. (2012). Primary stability of orthodontic mini-implants inserted into maxilla and mandible of swine. Oral. Surg. Oral. Med. Oral. Pathol. Oral. Radiol..

[15] Kuroda S., Sugawara Y., Deguchi T., Kyung H.-M., Takano-Yamamoto T. (2007). Clinical use of miniscrew implants as orthodontic anchorage: Success rates and postoperative discomfort. Am. J. Orthod. Dentofac. Orthop..

[16] Watanabe K., Mitchell B., Sakamaki T., Hirai Y., Kim D.-G., Deguchi T., Suzuki M., Ueda K., Tanaka E. (2021). Mechanical stability of orthodontic miniscrew depends on a thread shape. J. Dent. Sci..

[17] Wilmes B., Ottenstreuer S., Su Y.-Y., Drescher D. (2008). Impact of Implant Design on Primary Stability of Orthodontic Mini-implants. J. Orofac. Orthop. Kieferorthopadie.

[18] Mešić E., Muratović E., Redžepagić-Vražalica L., Pervan N., Muminović A.J., Delić M., Glušac M. (2021). Experimental & FEM Analysis of Orthodontic Mini-Implant Design on Primary Stability. Appl. Sci..

[19] Cozzani M., Nucci L., Lupini D., Dolatshahizand H., Fazeli D., Barzkar E., Naeini E., Jamilian A. (2020). The ideal insertion angle after immediate loading in Jeil, Storm, and Thunder miniscrews: A 3D-FEM study. Int. Orthod..

[20] Scribante A., Montasser M.A., Radwan E.S., Bernardinelli L., Alcozer R., Gandini P., Sfondrini M.F. (2018). Reliability of orthodontic minis-crews: Bending and maximum load of different Ti-6Al-4V titanium and stainless steel temporary anchorage devices (TADs). Materials.

[21] Kim G.-T., Jin J., Mangal U., Lee K.-J., Kim K.-M., Choi S.-H., Kwon J.-S. (2020). Primary Stability of Orthodontic Titanium Miniscrews due to Cortical Bone Density and Re-Insertion. Materials.

[22] da Cunha A.C., Marquezan M., Lima I., Lopes R.T., Nojima L.I., Sant'Anna E.F. (2015). Influence of bone architecture on the primary stability of different mini-implant designs. Am. J. Orthod. Dentofac. Orthop..

[23] Kang S.-Y., Yu J.-M., Kim H.-S., Lee J.-S., Yeon C.-M., Park K.-S., Choi S.-H., Lee S.-Y. (2020). Influence of Orthodontic Anchor Screw Anchorage Method on the Stability of Artificial Bone: An In Vitro Study. Materials.

[24] Barros S.E., Janson G., Chiqueto K., Garib D.G., Janson M. (2011). Effect of mini-implant diameter on fracture risk and self-drilling efficacy. Am. J. Orthod. Dentofac. Orthop..

[25] Gracco A., Giagnorio C., Incerti Parenti S., Alessandri Bonetti G.A., Siciliani G.S. (2012). Effects of thread shape on the pullout strength of miniscrews. Am. J. Orthod. Dentofac. Orthop..

[26] Tepedino M., Masedu F., Chimenti C. (2017). Comparative evaluation of insertion torque and mechanical stability for self-tapping and self-drilling orthodontic miniscrews—An in vitro study. Head Face Med..

[27] Lim S.-A., Cha J.-Y., Hwang C.-J. (2008). Insertion Torque of Orthodontic Miniscrews According to Changes in Shape, Diameter and Length. Angle Orthod..

[28] Radwan E.S., Montasser M.A., Maher A. (2018). Influence of geometric design characteristics on primary stability of orthodontic miniscrews. J. Orofac. Orthop. Kieferorthopadie.

[29] Migliorati M., Benedicenti S., Signori A., Drago S., Cirillo P., Barberis F., Biavati A.S. (2012). Thread shape factor: Evaluation of three different orthodontic miniscrews stability. Eur. J. Orthod..

[30] Sharma A., Maurya N.K., Singh Y., Singh N.K., Gupta S.K. (2020). Effect of design parameters on performance and emissions of DI diesel engine running on biodiesel-diesel blends: Taguchi and utility theory. Fuel.

[31] Ye Y., Yi W., Fan S., Zhao L., Yu Y., Lu Y., Yao Q., Wang W., Chang S. (2023). Effect of thread depth and thread pitch on the primary stability of miniscrews receiving a torque load: A finite element analysis. J. Orofac. Orthop..

[32] Kuroda S., Tanaka E. (2014). Risks and complications of miniscrew anchorage in clinical orthodontics. Jpn. Dent. Sci. Rev..

[33] Kitahara-Céia F.M., Assad-Loss T.F., Mucha J.N., Elias C.N. (2013). Morphological evaluation of the active tip of six types of orthodontic mini-implants. Dent. Press. J. Orthod..

[34] (2019). Standard Specification for Wrought 18Chromium-14Nickel-2.5Molybdenum Stainless Steel Bar and Wire for Surgical Implants (UNS S31673).

[35] (2016). Implants for Surgery—Metallic Materials—Part 1: Wrought Stainless Steel.

[36] (2021). Standard Specification for Rigid Polyurethane Foam for Use as a Standard Material for Testing Orthopaedic Devices and Instruments.

[37] (2017). Standard Specification and Test Methods for Metallic Medical Bone Screws.

[38] Topouzelis N., Tsaousoglou P. (2012). Clinical factors correlated with the success rate of miniscrews in orthodontic treatment. Int. J. Oral Sci..

[39] Papageorgiou S.N., Zogakis I.P., Papadopoulos M.A. (2012). Failure rates and associated risk factors of orthodontic miniscrew implants: A meta-analysis. Am. J. Orthod. Dentofac. Orthop..

[40] Jin J., Kim G.-T., Kwon J.-S., Choi S.-H. (2020). Effects of Intrabony Length and Cortical Bone Density on the Primary Stability of Orthodontic Miniscrews. Materials.

[41] Brinley C.L., Behrents R., Kim K.B., Condoor S., Kyung H.-M., Buschang P.H. (2009). Pitch and Longitudinal Fluting Effects on the Primary Stability of Miniscrew Implants. Angle Orthod..

[42] Suzuki E.Y., Suzuki B. (2011). Placement and removal torque values of orthodontic miniscrew implants. Am. J. Orthod. Dentofac. Orthop..

[43] Vaziri A.S., Naseri H., Nourisari M., Qaffari H., Kashaniaraqbidi M., Eslamiamirabadi R. (2015). The clinical success of self-tapping and self-drilling orthodontic minis-crews. Eur. J. Orthod..

[44] Yi J., Ge M., Li M., Li C., Li Y., Li X., Zhao Z. (2016). Comparison of the success rate between self-drilling and self-tapping miniscrews: A systematic review and meta-analysis. Eur. J. Orthod..

[45] Valeri C., Aloisio A., Quinzi V., di Stefano G., Marzo G. (2024). Characterizing orthodontic mini-screws in the hard palate of pigs: An experimental and finite-element study. Heliyon.

[46] Sousa-Santos C., Sousa-Santos S., Mendes J., Coelho C., Aroso C., Sousa-Santos P., Mendes J.M. (2024). The Influence of the Diameter of Orthodontic Mini-Implants on Primary Stability: Bending Tests—An In Vitro Study. Materials.

[47] Do T.V., Hsu Q.C., Chen P.H., Chen Y.L. (2016). Study on the Performance of Orthodontic Self-Drilling Correction Screw of Ti6Al4V and Stainless 316L. Mater. Sci. Forum.

[48] Boukraa M., Chekifi T., Lebaal N. (2022). Friction Stir Welding of Aluminum Using a Multi-Objective Optimization Approach Based on Both Taguchi Method and Grey Relational Analysis. Exp. Tech..

[49] Hwang J.-R., Zheng J.-Y., Kuo P.-C., Huang C.-D., Fung C.-P. (2022). Process Optimization of Inconel 718 Alloy Produced by Laser Powder Bed Fusion. Metals.

[50] Phan T.-D., Do T.-V., Pham T.-L., Duong H.-L. (2020). Optimization of cutting parameters and nanoparticle concentration in hard milling for surface roughness of JIS SKD61 steel using linear regression and Taguchi method. Lect. Notes Netw. Syst..

